# 

**Published:** 1819-09

**Authors:** 


### MONTHLY CATALOGUE OF MEDICAL BOOKS.

The Accoucheur's Vade-mecum; by Joseph Hopkins, M.D. 2 vols. 12mo.

Practical Researches on the Nature, Cure, and Prevention of Gout, in all its open and concealed Forms; by James Johnson, Esq. 8vo.

A Medical Dictionary; containing an Explanation of the Terms in Surgery, Medicine, Midwifery, Anatomy, &c. By J. J. Watt, Surgeon. 12mo. 2d edit.

A Series of Anatomical, Physiological, and Surgical Lectures, on the Vascular System. By James Wilson, F.R.S. Professor of Anatomy and Surgery, &c. 8vo.

An Essay on Chemical Analysis. By John George Children, F.R.S.L. & E.F.A.S. &c. 8vo. boards, 16s.

Pathological and Practical Remarks on Ulcerations of the Genital Organs. By James Evans, Surgeon, 57th Regiment. 8vo. boards, 5s. 6d.

A Treatise on the Nature and Cure of Gout and Rheumatism. By Charles Scudamore, M.D. 8vo. boards, 1l.

Practical Illustrations of Typhous Fever. By John Armstrong, M.D. Third edition. 8vo. boards, 14s.

A History of the High Operation for the Stone, by an Incision above the Pubis. By J. C. Carpue, F.R.S. 8vo. boards, 8s. 6d.

